# Urban Advanced Mobility Dependability: A Model-Based Quantification on Vehicular Ad Hoc Networks with Virtual Machine Migration

**DOI:** 10.3390/s23239485

**Published:** 2023-11-28

**Authors:** Luis Guilherme Silva, Israel Cardoso, Carlos Brito, Vandirleya Barbosa, Bruno Nogueira, Eunmi Choi, Tuan Anh Nguyen, Dugki Min, Jae Woo Lee, Francisco Airton Silva

**Affiliations:** 1 Coordination of the Information Systems Course, Technical College of Teresina (CSHNB), Federal University of Piauí (UFPI), Picos 64049-550, Piauí, Brazil; luis.e@ufpi.edu.br (L.G.S.); israel.araujo@ufpi.edu.br (I.C.); carlosvictor@ufpi.edu.br (C.B.); vandirleya.barbosa@ufpi.edu.br (V.B.); faps@ufpi.edu.br (F.A.S.); 2 Instituto de Computação, Federal University of Alagoas (UFAL), Maceió 57072-900, Alagoas, Brazil; bruno@ic.ufal.br; 3 School of Software, College of Computer Science, Kookmin University, Seoul 02707, Republic of Korea; emchoi@kookmin.ac.kr; 4 Konkuk Aerospace Design-Airworthiness Research Institute (KADA), Konkuk University, Seoul 05029, Republic of Korea; 5 Department of Computer Science and Engineering, College of Engineering, Konkuk University, Seoul 05029, Republic of Korea; 6 Department of Aerospace Information Engineering, Konkuk University, Seoul 05029, Republic of Korea

**Keywords:** Vehicular Ad Hoc Networks (VANETs), dependability modeling, Stochastic Petri Nets (SPN), virtual machine migration, network reliability and availability

## Abstract

In the rapidly evolving urban advanced mobility (UAM) sphere, Vehicular Ad Hoc Networks (VANETs) are crucial for robust communication and operational efficiency in future urban environments. This paper quantifies VANETs to improve their reliability and availability, essential for integrating UAM into urban infrastructures. It proposes a novel Stochastic Petri Nets (SPN) method for evaluating VANET-based Vehicle Communication and Control (VCC) architectures, crucial given the dynamic demands of UAM. The SPN model, incorporating virtual machine (VM) migration and Edge Computing, addresses VANET integration challenges with Edge Computing. It uses stochastic elements to mirror VANET scenarios, enhancing network robustness and dependability, vital for the operational integrity of UAM. Case studies using this model offer insights into system availability and reliability, guiding VANET optimizations for UAM. The paper also applies a Design of Experiments (DoE) approach for a sensitivity analysis of SPN components, identifying key parameters affecting system availability. This is critical for refining the model for UAM efficiency. This research is significant for monitoring UAM systems in future cities, presenting a cost-effective framework over traditional methods and advancing VANET reliability and availability in urban mobility contexts.

## 1. Introduction

Vehicular Ad Hoc Networks (VANETs) represent a significant advancement in wireless communication technology. These networks are pivotal in enhancing vehicular connectivity, thereby fostering a safer and more efficient environment for all traffic participants. GSMA estimates indicate that approximately 20% of the global vehicular fleet, estimated at 1.5 billion, are internet-connected, contributing substantially to data generation [1]. Projections suggest that by 2027, there will be an annual growth rate of around 17%, resulting in 367 million connected vehicles.

Typically, a VANET incorporates a static infrastructure component, known as a Roadside Unit (RSU), positioned alongside thoroughfares. Vehicles interface with this infrastructure through an onboard unit (OBU) [2]. It is presumed that each vehicle is outfitted with sensors to collect environmental data. The OBU processes these data and engages in communication with other vehicles or RSUs, either directly or indirectly. Additionally, RSUs have the capability to connect to the internet, thereby facilitating vehicular access to various services [3].

VANETs find application in diverse traffic-related domains, encompassing areas such as network security [4], traffic management [5], and parking space optimization [6]. Nonetheless, managing the Quality of Service (QoS) within VANETs poses a multifaceted and critical challenge. Key challenges include ensuring network availability and reliability, addressing latency, and managing traffic, as well as grappling with issues of poor connectivity, limited flexibility, and scalability constraints. The physical distance from Edge processing and storage centers can also introduce considerable communication delays [7,8,9].

The literature review reveals a paucity of studies employing Stochastic Petri Nets (SPN) in the context under consideration in this research. The proposed model incorporates crucial factors such as availability and reliability, utilizing VM migration across the network to link various RSUs and implementing an Edge Computing-based data processing system. This model facilitates a comprehensive analysis of the key parameters, aiming to refine architectures to address the integration challenges of VANETs with Edge Computing. The principal contributions of this research are as follows:

- Development of an SPN model to assess the reliability and availability of VANET-based VCC architectures, factoring in stochastic elements to emulate realistic scenarios. This aims to enhance the robustness of vehicular network environments, ensuring dependable and consistent performance.
- Execution of case studies using the proposed models, offering a blueprint for other researchers in applying these models. These case studies focus on identifying and analyzing primary parameters influencing system availability, providing preliminary insights into the critical variables affecting system reliability, and facilitating enhancements and optimizations.
- Conducting a sensitivity analysis of the SPN model components, identifying parameters with significant influence on system availability. This analysis enhances the understanding of the model and aids in its optimization.

The structure of this work is organized as follows: Section 2 introduces essential concepts foundational to this study. Section 3 describes the architecture forming the basis of the model. Section 4 details the developed SPN model. Section 5 presents two sensitivity analyses performed on the SPN model. Section 6 elaborates on the outcomes of the case study. Finally, Section 7 concludes the paper and outlines directions for future work.

## 2. Background

This section succinctly delineates the core concepts fundamental to this research, primarily focusing on SPNs. An elucidation of experimental design and sensitivity analysis will follow. These concepts are critical for comprehending the methodologies and techniques employed in the formulation of this article. Understanding these foundational principles is essential for appreciating the intricacies of the proposed models and analyses within the context of this study. Related works of the above discussions are provided in Table 1.

### 2.1. Stochastic Petri Nets

A Petri Net is a combined graphical and mathematical representation that effectively models systems and processes undergoing continuous changes and concurrent operations. This modeling is particularly valuable for systems characterized by simultaneous multi-action scenarios [10]. The present study employs a sophisticated form of Petri Nets, termed SPNs, which are distinguished by their ability to incorporate randomness and probabilistic behaviors [11]. SPNs are comprised of three primary elements that delineate the system’s states or conditions: transitions (denoting potential system events or actions), tokens (symbolizing entities within the system), and each token’s potential association with a specific resource [12].

The functionality of SPNs hinges on two essential types of connections: (I) Input Arcs, which are preconditions for triggering transitions, necessitating specific tokens’ presence at designated locations; and (II) Output Arcs, which dictate the subsequent transferal of tokens upon a transition’s activation [13]. Transitions in SPNs are categorized into timed transitions, obeying stochastic distributions [14], and immediate transitions that occur instantaneously upon activation. Additionally, inhibitor arcs play a pivotal role in controlling token flow between locations, with tokens being allocated to particular places within the system.

**Figure 1:**
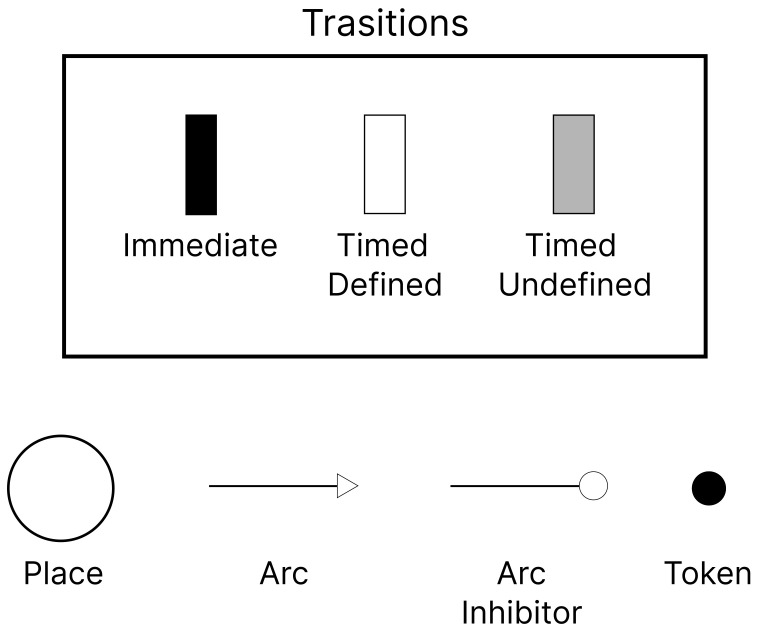
SPN components.

Crucially, SPNs utilize guard conditions to define the specific prerequisites for transition activations. These conditions often incorporate random variables, thus introducing a probabilistic dimension. The fulfillment of these guard conditions triggers transitions according to predefined rate functions, effectuating a change in the system’s state [15]. Figure 1 visually explicates the core components of an SPN model, providing a comprehensive understanding of its structure and functionality.

### 2.2. Sensitivity Analysis with DoE

Design of Experiments (DoE) is an extensively utilized methodology in research and development for enhancing processes, products, and systems. It entails the meticulous planning and execution of controlled experiments to gather pertinent and substantial data [16]. The initial step in DoE involves defining the experiment’s objective, followed by identifying the variables or factors that could influence the outcome. Subsequently, an experimental plan is formulated, which includes determining the levels for each factor and designing the experiments to extract significant insights. The execution of the experiment is aligned with this plan, leading to the collection and statistical analysis of the results.

DoE equips system designers with the ability to discern the most influential variables, understand their interactions, and fine-tune the conditions to achieve optimal outcomes with minimal experimental iterations [17]. Complementing DoE, sensitivity analysis serves as a pivotal technique for examining how variations in the input parameters or model attributes influence the outputs or results [18]. This analysis is essential for assessing the robustness of a system or model in the face of uncertainties or changes in parameters [19].

Through sensitivity analysis, it becomes feasible to identify which variables significantly affect outputs and which have lesser impacts, thereby guiding the allocation of resources and efforts towards efficient system optimization. Both DoE and sensitivity analysis play crucial roles in research, engineering, and strategic decision making, providing pathways to more effective solutions, resource and time conservation, and the acquisition of valuable insights for the enhancement of processes and systems. This efficiency is achieved as they necessitate fewer experiments to glean significant information [20,21].

**Table 1:** Related works.

Reference	Contribution	Assessment Method	Metrics	Multiple RSUs	Sensitivity Analysis
[22]	A performance modeling of media access control (MAC).	Simulation	Performance	No	No
[23]	Threat-Oriented Authentication Approach for Secure Communication.	Simulation	Performance	No	No
[24]	Modeling that integrates the transmission of the 802.11p system and the queuing process.	Simulation	Performance	No	No
[25]	A mobile agent-based information dissemination scheme in the VANET environment.	Simulation	Performance	No	No
[26]	A mobile agent migration mechanism based on location simulation experiments in the VANET environment.	Simulation	Performance	No	No
[27]	A TCP Context Migration Scheme (TOMS) method for enhancing data services in vehicular networks.	Simulation	Performance	Yes	No
[28]	Detection of anomalies, loss of messages with conventional and VEC techniques.	Simulation	Availability	Yes	No
[29]	A container-based virtualization and live migration framework for the in-vehicle ad hoc network.	Measurement	Performance	Yes	No
[30]	Provide a classification of security requirements, characteristics and security challenges.	Measurement	Does not have	Yes	No
[31]	A seamless handover system in a software- defined network (SDN) framework.	Measurement	Performance	Yes	No
[32]	BaaS (Broadcast as a Service) transmission is proposed for VANET to disseminate data efficiently to network vehicles using cloud computing.	Measurement	Performance	Yes	No
[33]	It presents a model for the connectivity patterns of chains of vehicles traveling on a highway.	Markov Model	Availability	No	No
[34]	Analytical model based on Stochastic Petri Net (SPN) theory for assessment of Vehicular Ad Hoc Network infrastructures.	SPN Model	Performance	No	No
[35]	Use SDN to improve the allocation and migration of microservices in Vehicular Fog Networks (VFN).	Measurement	Performance	Yes	No
This work	Modeling an architecture with multiple RSUs and migration to assess system availability.	SPN Model	Availability	Yes	Yes

#### 2.2.1. Simulation-Based Methods

In the initial category of research, simulation served as the primary method for evaluation. The study by [22] focused on assessing the performance of Medium Access Control (MAC) protocols within VANETs. Ref. [23] introduced a threat-oriented authentication strategy designed to bolster secure communication in vehicle-to-vehicle (V2V) and vehicle-to-infrastructure (V2I) interactions, utilizing a combination of encryption keys. The research conducted by [24] highlighted deficiencies in the 802.11 system, particularly the absence of backoff and binary exponential retransmission mechanisms, which were found to adversely affect the QoS during periods of intense traffic.

Further, Ref. [25] proposed an innovative mobile agent migration mechanism. This mechanism, rooted in network location analytics, was employed to simulate a VANET environment, exploring its practical applications. In parallel, the studies by [26,27] utilized the TCP Context Migration Scheme (TOMS), a novel approach aimed at enhancing data services within vehicular networks. This scheme entailed the proactive establishment of TCP connections, managed by a mobile TCP proxy that assumed the role of a cluster leader.

Lastly, the investigation by [28] delved into the application of Vehicle Edge Computing (VEC) alongside conventional anomaly detection techniques. This approach was targeted at identifying and quantifying message loss, thereby evaluating the extent of fault coverage within these networked systems.

#### 2.2.2. Measurement-Based Methods

In the second category, the selected studies employed measurement as their primary evaluation methodology. Ref. [29] developed a container-based virtualization architecture, facilitating dynamic migration within ad hoc vehicular networks, particularly in VANETs. This innovation aimed to enhance flexibility and responsiveness in these networks. Meanwhile, Ref. [30] undertook the task of classifying security requirements, identifying key characteristics, and delineating the challenges related to security within similar VANET scenarios.

In another significant contribution, Ref. [31] introduced a seamless transition system that leverages the capabilities of SDN and Media Independent Handover (MIH). This system was designed to dynamically modify the topology of VANETs, enhancing their adaptability and efficiency. Additionally, Ref. [32] proposed a novel concept termed Broadcast as a Service (BaaS), specifically tailored for VANETs. This solution aimed to efficiently disseminate data across networked vehicles utilizing cloud computing technologies. Lastly, the work of Ref. [35] applied SDN to improve the management and migration of microservices in Vehicular Fog Networks (VFNs), taking into account the dynamic nature of vehicular nodes.

#### 2.2.3. Modeling-Based Methods

The third classification encompasses studies that utilized modeling as their core evaluation technique. The research of [33] presented a model to describe the connectivity patterns among vehicles on highways. This model is crucial for the development of protocols and applications in VANETs, tailored to their specific connectivity traits. Similarly, [34] adopted an analytical approach rooted in SPN theory. This approach was used to assess the infrastructure of VANETs, taking into consideration the mobility of the network and its inherent limitations. Notably, this study modeled the service fees of RSUs using exponential distributions.

#### 2.2.4. Contributions of This Work in Relation to Others

This study introduces an SPN model that evaluates the impact of VM migration across multiple RSUs within vehicular networks. A review of the literature reveals the scarcity of studies addressing this specific scenario within the VANET context. The adoption of such an approach is economically advantageous, as it enables the analysis of availability and reliability without necessitating a physical infrastructure for testing.

The review further indicates that systems modeling, as employed in this study, provides a more predictive and comprehensive understanding compared to measurement and simulation methods. This is achieved by simplifying the representation of critical system elements. In contrast, measurement and simulation tend to rely on observational data, which may not fully encapsulate the complexity of the system. Most of the reviewed studies focused primarily on performance evaluation, with limited attention to metrics such as availability, reliability, or downtime. Moreover, there is a noticeable gap in studies exploring the cooperation between multiple RSUs and the use of sensitivity analysis to determine the impact of Mean Time To Failure (MTTF) and Mean Time To Repair (MTTR) parameters on system performance.

## 3. Evaluated Architecture

**Figure 2:**
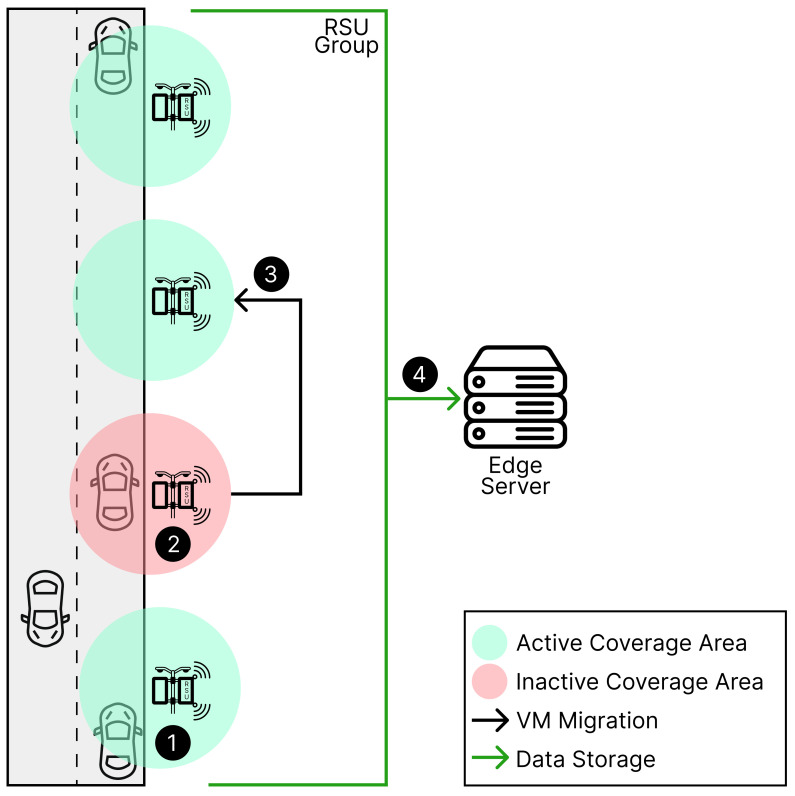
Base architecture.

In this section, the envisaged architecture for integrating VANETs is elucidated. The foundational scenario, as illustrated in Figure 2, encompasses an array of RSUs, with their respective coverage zones depicted as green and red circles. The operational dynamics of this architecture are as follows:

- (1) *Active RSU Coverage and Vehicle Interaction*: Vehicles in transit enter the coverage area of active RSUs (represented by green circles), wherein these RSUs facilitate communication and gather data from the vehicles.
- (2) *Response to RSU Failure*: In the event of an RSU malfunction, leading to a disruption in data collection, a contingency protocol is activated.
- (3) *VM Migration for Uninterrupted System Availability*: To ensure continued system functionality, an allocation of data from VMs is performed that will be transferred to the subsequent RSU within the network after an RSU fails.
- (4) *Data Management*: Subsequent to collection, all data are transmitted to an Edge Server for storage and further processing.

The RSUs in this architecture are equipped with advanced communication technologies, potentially including 5G [36] or Lora [37], enabling interaction with vehicular systems. These units are strategically placed along roadways to form a dependable communication infrastructure, essential for the efficient operation of the VANET system. This arrangement guarantees a seamless data flow and operational continuity of the system, even amidst individual RSU failures, thereby augmenting the reliability and resilience of the VANET infrastructure.

In this segment, the technical composition of the VANETs is detailed, emphasizing the integration of On-Board Units (OBUs) in each vehicle. These OBUs are imbued with communication capabilities, enabling the transmission and reception of messages. Additionally, they are outfitted with GPS tracking devices, facilitating the sharing of precise, real-time locational data [38]. The infrastructure is based on the assumption that all RSUs maintain a connection to a private Edge server via a high-speed wireless link, such as 5G; 5G offers faster data transmission speeds and reduced latency, key features for system efficiency [39].

The selection of these technologies plays a significant role in influencing the overall system availability [40]. The choice of communication technology emerges as a vital consideration in the model’s design process. The architecture’s design is inherently scalable, allowing for the integration of a variable number of RSUs to meet the specific requirements of the deployment area. The depicted scenario in the figure showcases four groups of RSUs, but this configuration can be dynamically adjusted to suit varying demands.

A key attribute of this architecture is its fault detection capability in RSUs. When an RSU is compromised, indicated by red in Figure 2, it triggers the migration of VMs to the next cluster of RSUs. This migration is essential for maintaining operational continuity and ensuring system availability, thereby minimizing disruptions in communication and data processing.

Data processing in this system is executed at the Edge (Edge Computing), which enhances communication efficiency and reduces latency. The proximity of RSUs to the vehicles permits a portion of the data processing to occur locally, thus enhancing the system’s real-time responsiveness. The proposed architecture aims to establish an effective communicative link between vehicles and cloud infrastructure, with a strong focus on reliability, scalability, and the migration of VMs to guarantee uninterrupted operations. The ensuing section will delve into the model used to assess the reliability and availability of this system.

## 4. Proposed Model

This section delineates the models applied in this study, which are constructed based on the architecture outlined in the preceding section. It further details the reliability and representative availability models in scenarios both with and without the implementation of migration strategies. All the models and simulations in this study were conducted using the Mercury Tool [41].

### 4.1. System Reliability Model

**Figure 3:**
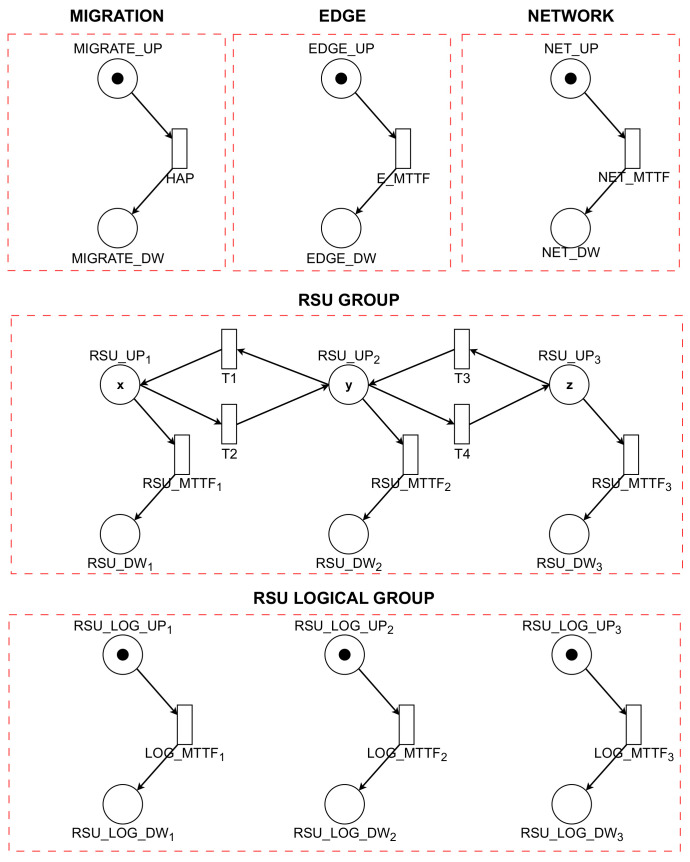
Reliability model.

**Figure 4:**
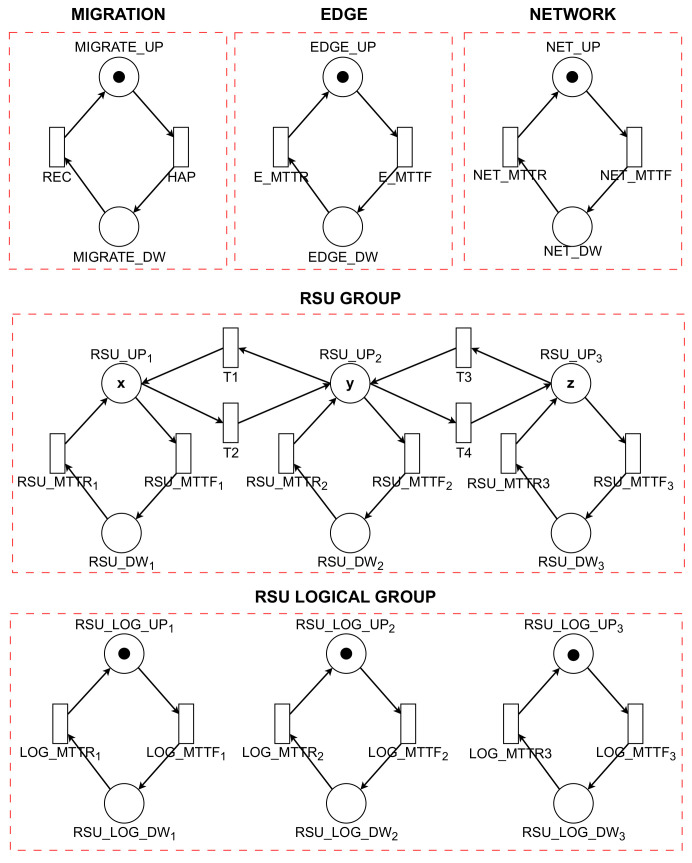
Availability model with migration.

The model for analyzing the reliability of VANETs is depicted in Figure 3. Within this context, reliability is defined as the conditional probability that a system will continue functioning over a time interval [0, t], provided that it was operational at the inception of this interval (t = 0). The presented model (Figure 3) bears resemblance to the model in Figure 4, with a notable distinction: it excludes the MTTR transitions that would facilitate the recovery of components in the event of a failure. This exclusion is a critical aspect, as it directly impacts the system’s ability to self-recover post-failure, thereby influencing the overall reliability assessment of the VANET system under study.

In the RSU GROUP within the VANET system, the RSUs can host varying numbers of VMs, symbolized by the variables x, y, and z. The reliability of the model under consideration can be quantitatively assessed using Equation (1). This metric is defined as the complement of the probability of failure of any component in the system. Specifically, it is represented as one minus the probability that the RSU Group, RSU Logical Group, Network, and Edge components play all operations simultaneously:

(1) $$\begin{array}{cc} R=1-P( & (\#EDGE\_U>0)\;\mathrm{AND}\;(\#\mathrm{NET}\_\mathrm{UP}>0)\;\mathrm{AND}\;\\ \; & ((\#\mathrm{RSU}\_\mathrm{UP}1+\#\mathrm{RSU}\_\mathrm{UP}2)+\#\mathrm{RSU}\_\mathrm{UP}3)=(n\cdot \mathrm{TOKENS})\;\mathrm{AND}\;\\ \; & \left(\#\mathrm{RSU}\_\mathrm{LOG}\_\mathrm{UP}1=1\;\mathrm{OR}\;\#\mathrm{RSU}\_\mathrm{LOG}\_\mathrm{UP}2=1\;\mathrm{OR}\;\#\mathrm{RSU}\_\mathrm{LOG}\_\mathrm{UP}3=1)\right) \end{array}$$

In this model, the variable “ *P*” is utilized to determine the probability that the system will become unavailable or fail. By applying this equation, it is possible to generate a curve that effectively illustrates how the system’s reliability diminishes over time. This curve is a crucial analytical tool, as it provides a visual representation of the system’s reliability, enabling the identification of trends and potential vulnerabilities over a specified time frame. Such an analysis is vital for understanding the robustness of the system and for making informed decisions regarding maintenance, upgrades, or other interventions to enhance the system’s reliability.

### 4.2. Availability Model with Migration

Table 2 systematically details the elements of the availability model, employing tokens to represent the count of operational VMs within each RSU. In this model, the transitions labeled *RSU_MTTF* and *RSU_MTTR* are integral for facilitating the exchange of information among different RSU groups via VMs. For the effective operation of an RSU Group, it is imperative that the quantity of tokens in the *RSU_UP* state aligns with the initially set value.

The availability of an RSU is contingent upon the presence of tokens in the *RSU_LOG_UP* state and their absence in the *RSU_LOG_DW* state. Concurrently, the operational state of the NETWORK is indicated by the distribution of tokens: tokens in the *NET_UP* state signify an active network, while those in *NET_DW* denote an inactive state. This model underscores the reliance on token distribution for depicting the dynamic status of each RSU and the overall network, thus providing a comprehensive view of the system’s availability and the efficacy of the migration strategy.

The transitions labeled *NET_MTTF* and *NET_MTTR* are pivotal in the management of the network’s operational dynamics. Concurrently, the functioning of the EDGE component relies on the *E_MTTF* and *E_MTTR* transitions, which govern the activation (*EDGE_UP*) and deactivation (*EDGE_DW*) states. The migration process, a key element for ensuring uninterrupted system operation, is regulated through *MIGRATE_UP* (activation) and *MIGRATE_DW* (deactivation) markers. These markers facilitate the transfer of VMs between RSUs, an action critical for maintaining system availability.

Figure 4 presents the proposed SPN model, which is constructed based on the previously outlined scenario. This model encompasses various components such as RSU GROUP, RSU LOGICAL GROUP, NETWORK, EDGE, and MIGRATION. Each of these components is associated with metrics like MTTF and MTTR, which are instrumental in evaluating the availability and reliability of systems and their individual components.

The operational status of the VMs in each RSU is indicated by the presence of tokens in the *RSU_UP* and *RSU_DW* states. Here, *RSU_UP* denotes active RSUs, while *RSU_DW* represents inactive ones. The transitions between the active and inactive states of each Road Service Unit (RSU) are controlled by the *RSU_MTTF* and *RSU_MTTR* transitions. Within the RSU Group, each unit contributes to the collective functionality by sharing information via VMs. For the RSU Group to function effectively, it is essential that the number of tokens in *RSU_UP* aligns with its pre-established initial value.

Table 3 illustrates the implementation of guard conditions in the transitions T1, T2, T3, and T4, which are situated between the RSUs designated as UP1, UP2, and UP3. These guard conditions are essential for regulating the migration of VMs in scenarios where a logical component of the RSU becomes unavailable. When this logical component is subsequently reactivated and resumes normal operation, the previously migrated VMs are reintegrated into their original RSU. This mechanism of migration and reintegration is pivotal in ensuring the seamless and continuous functioning of the system, as it provides a dynamic response to temporary outages or disruptions within individual RSUs, thereby maintaining overall system integrity and operational continuity.

The transition T2 is activated by the guard condition #*RSU_LOG_DW*1=1, which denotes a critical event indicative of the instability or inactivation of RSU 1’s logical component. This event initiates the process for VM migration from the compromised RSU. The procedure begins by verifying the operational status of the subsequent RSU, indicated by #*RSU_LOG_DW*2=0. Should this RSU be operational, the migration of VMs is executed accordingly. Subsequently, in the event of a recovery and reactivation of the initially failed RSU, the previously migrated VMs are reintegrated into it. In a parallel scenario, transition T4 is invoked when #*RSU_LOG_Dw2*=1, a condition signaling the deactivation of the logical component of RSU 2. This state necessitates the migration of VMs from RSU 2 to another operational unit, thereby ensuring the continuity of system functionality.

The availability of the model with migration is quantified using Equation (2). This equation computes the probability that the RSU Group, RSU Logical Group, Network, and Edge components are all operational concurrently. In this context, ‘P’ denotes the probability, while ‘TOKENS’ refers to the number of tokens present in a specific state or place within the model. This approach to calculating availability is crucial for assessing the effectiveness of the migration strategy in maintaining continuous operation of the system, even in the face of individual component failures or disruptions. The inclusion of these probabilistic measures provides a comprehensive understanding of the system’s resilience and its ability to sustain uninterrupted service through dynamic VM migration processes:

(2) $$\begin{array}{cc} A=P( & \left(\#EDGE_{U}>0\right)\;\mathrm{AND}\;\left(\#\mathrm{NET}\_\mathrm{UP}>0\right)\;\mathrm{AND}\;\\ \; & ((\#\mathrm{RSU}\_\mathrm{UP}1+\#\mathrm{RSU}\_\mathrm{UP}2)+\#\mathrm{RSU}\_\mathrm{UP}3)=(n\cdot \mathrm{TOKENS})\;\mathrm{AND}\;\\ \; & \left(\#\mathrm{RSU}\_\mathrm{LOG}\_\mathrm{UP}1=1\;\mathrm{OR}\;\#\mathrm{RSU}\_\mathrm{LOG}\_\mathrm{UP}2=1\;\mathrm{OR}\;\#\mathrm{RSU}\_\mathrm{LOG}\_\mathrm{UP}3=1)\right) \end{array}$$

### 4.3. SPN Availability Model: Non-Migration Framework

**Figure 5:**
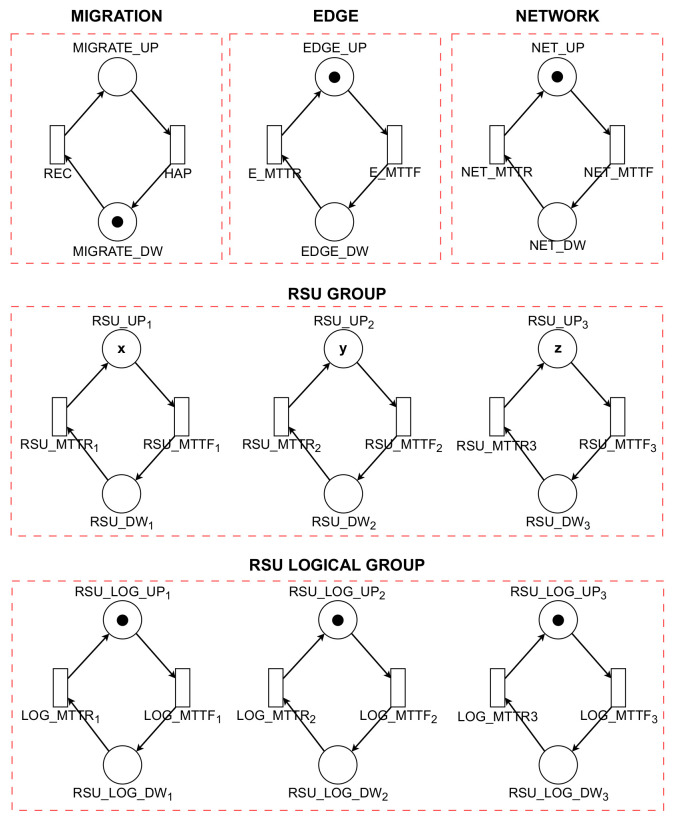
Availability model without migration.

The depicted SPN availability model in Figure 5 delineates the system’s functionality in the absence of migration capabilities. This model parallels its counterpart incorporating migration, encompassing components such as MIGRATION, EDGE, NETWORK, RSU GROUP, and RSU LOGICAL GROUP. Integral to each component is an MTTF and a singular MTTR, with the exception of MIGRATION, which is characterized by the Happened attribute (HAP). This attribute simulates potential disasters or system instabilities, leading to the activation of *MIGRATE_DW*, thereby deactivating migration processes.

A notable deviation in this non-migratory model is the omission of transition mechanisms within the RSU GROUP, precluding inter-RSU migration. The activation of the recovery process (REC) is contingent upon the RSU GROUP configuration, which facilitates the identification of operational RSUs via the tokens in *RSU_UP*. The symbol ‘N’ signifies the possibility of multiple tokens within each RSU, with the quantity of tokens representing the number of operational RSUs.

Conversely, the *RSU_DW* token count reflects the number of non-operational RSUs. The transitions *RSU_MTTF* and *RSU_MTTR* govern the oscillation between the active and inactive states of individual RSUs. Network functionality is ensured when a token resides in *NET_UP* (active network), and it becomes non-functional with a token in *NET_DW* (inactive network). The transitions *NET_MTTF* and *NET_MTTR* regulate these state changes.

Similarly, the cloud’s operational status is indicated by the presence of a token in *EDGE_UP*, and its non-operational status is signified by a token in *EDGE_DW*. The transitions *EDGE_MTTF* and *EDGE_MTTR* are instrumental in toggling between the cloud’s active and inactive states.

## 5. Sensitivity Analysis

This research utilizes a Design of Experiments (DoE) approach in conjunction with SPN modeling to derive comprehensive insights into the performance of Edge Computing systems. The variables and their respective levels are consistently applied across models, both incorporating and excluding migration. This methodological consistency is critical to ascertain which variable combinations exert the most significant impact on the system. By exploring these variable combinations across different scenarios, the study robustly underpins the optimization strategies proposed for enhancing the system’s availability and reliability.

### 5.1. Sensitivity Analysis of the System Incorporating Migration

The sensitivity analysis was conducted using the experimental setup illustrated in Figure 2, with a primary focus on the time interval preceding system failure. Table 4 presents a comprehensive enumeration of the variables considered in the Design of Experiments (DoE). This enumeration includes detailed descriptions of each factor and specifies their respective levels. The factors assessed are (a) *EDGE_F*, (b) *NET_F*, (c) *RSU_F*, (d) *RSU_R*, and (e) *LOG_F*. For each factor, evaluations were conducted at both high and low settings to ascertain their impact on the overall system performance. The specific configurations for these factors, as applied in the various experimental scenarios, are thoroughly delineated in the aforementioned table.

Table 5 comprehensively catalogs the permutations of factor levels employed in the simulations designed to assess their impact on system availability within an Edge Computing framework. Each row in the table represents a distinct amalgamation of factor values, specifically *EDGE_F*, *NET_F*, *RSU_F*, *RSU_R*, and *LOG_F*. The terminal column of this table quantifies the system availability corresponding to each unique factor combination. This tabulation effectively encapsulates the outcomes of the DOE simulations, facilitating the identification of factor combinations that either significantly influence or minimally affect the system’s availability. The structured presentation of these results serves as a pivotal resource for comprehending the dynamics influencing system performance and the relative importance of various system components.

**Figure 6:**
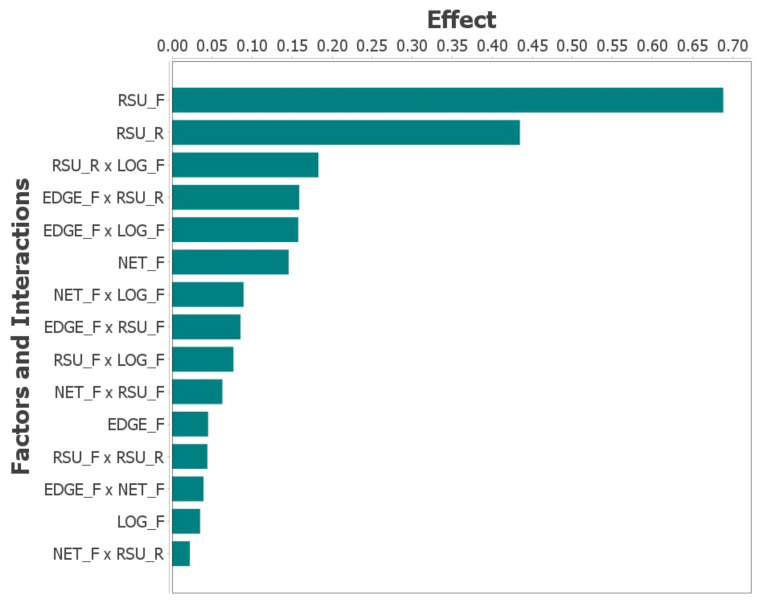
Impact of Different Factors on the System with Migration.

The graph depicted in Figure 6, illustrating the effects of a DOE with migration, provides pivotal insights into the elements most influential on system availability within an Edge Computing environment. Foremost, the MTTF of the physical RSU emerges as the paramount factor, with its impact value approximating 0.70. This underscores the criticality of physical infrastructure reliability in the overall system performance.

Following this, the MTTR of the physical components of RSU, with values oscillating between 0.40 and 0.45, is identified as another crucial determinant in minimizing system failures. This finding accentuates the significance of efficient repair processes in maintaining system integrity.

The interaction between the MTTR of the physical RSU and the MTTF of the logical RSU is also noted to exert a substantial influence on availability. In addition, the chart delineates factors with comparatively lower impacts, such as the interplay between the MTTF of the Edge component and the MTTF of the Network, the MTTF of the logical components, and the interaction between the MTTF of the Network and the MTTR of the physical RSU, each registering impact values below 0.05. While these elements are deemed less consequential in the present analysis, they could acquire greater significance in certain specific scenarios, suggesting the need for a nuanced understanding of different operational contexts in Edge Computing systems.

**Figure 7:**
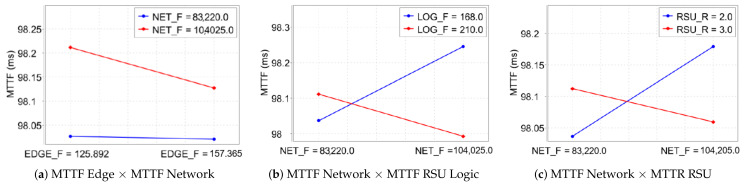
Interaction between factors in the system with migration.

Figure 7 elucidates the intricate interactions between various factors in a migration-inclusive scenario and their collective impact on system availability as assessed through a DOE approach.

In Figure 7a, the interaction between the Mean MTTF of the Edge component and the MTTF of the Network is analyzed. A notable decrease in system availability is observed when the MTTF of the Edge (157.0) is juxtaposed with the Network’s MTTF (98.3). This observation is indicative of the system’s heightened sensitivity to fluctuations in these parameters, underscoring the critical nature of their balance for optimal system performance.

In Figure 7b, the dynamics between the MTTF of the Network and the MTTF of the logical RSU component are examined. An inverse relationship is discerned here: an escalation in the MTTF of the Edge (from 168.0 to 210.0) correspondingly diminishes the MTTF of the logical RSU. This pattern exemplifies the complex interdependencies within the system, where enhancing the reliability of one component may inversely affect another, thereby impacting overall system availability.

In Figure 7c, the analysis is centered on the interplay between the MTTF of the Network and the MTTR of the physical RSU. An observed increase in the Network’s MTTF, along with an enhancement to 210 in the MTTF of the physical RSU component, indicates a notable enhancement in the overall system performance. This result underscores the critical importance of an integrated assessment of both failure and repair durations within the Network and physical components of the RSU. Such a holistic approach is essential for optimizing system availability. These insights collectively contribute to a deeper understanding of system reliability in Edge Computing environments, highlighting the need for a thorough evaluation of the interactions between various system components to achieve optimal system performance.

### 5.2. Analysis of System Sensitivity in the Absence of Migration

**Figure 8:**
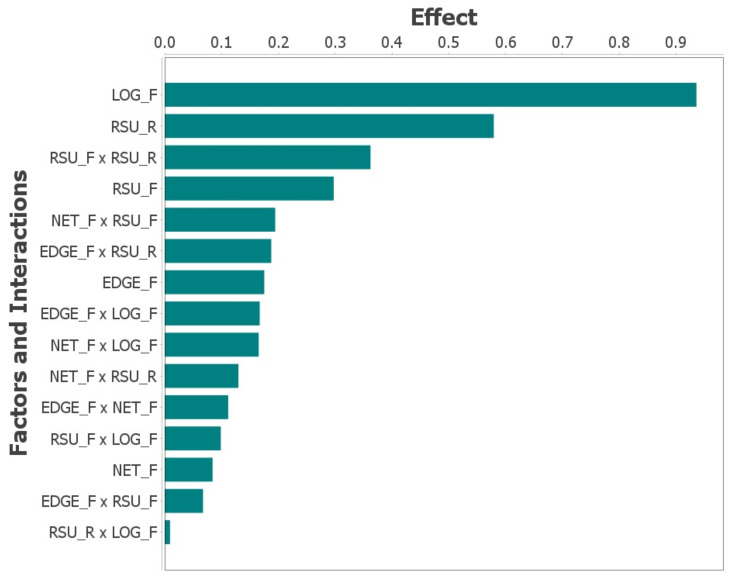
Impact of different factors on the system without migration.

The effect graph in Figure 8, derived from the DOE conducted without the migration feature, elucidates the principal factors impacting system availability in an Edge Computing context. The graph reveals that the MTTF of the logical RSU holds paramount significance, as indicated by its value exceeding 0.90. This underscores the critical role of the reliability of the logical RSU in the overall system.

Secondarily, the MTTR of the RSU physical components also emerges as a consequential factor, exhibiting values around 0.60. This finding emphasizes the importance of efficient repair mechanisms in mitigating system downtime during failures.

Other factors, though less influential, still contribute to the system’s performance. These include the MTTF of the Network and the interactions between the MTTF of the Edge component and the MTTF of the logical RSU. Additionally, the interplay between the MTTR of the physical RSU and the MTTF of the logical RSU, each with impact values below 0.1, also bears significance. These findings are instrumental in enhancing system reliability, particularly in scenarios where migrating VMs between RSUs is not feasible. They inform strategic resource allocation decisions and guide interventions aimed at bolstering the overall performance of Edge Computing systems in non-migratory environments. This nuanced understanding of the relative impact of various system components and their interactions is essential for targeted improvements in system robustness and reliability.

**Figure 9:**
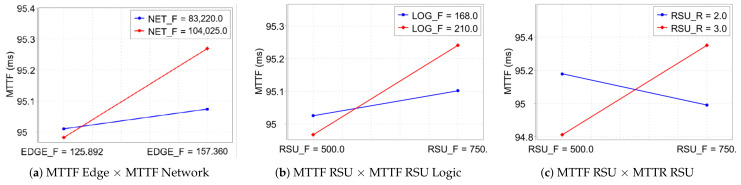
Interaction between factors in the system without migration.

Figure 9 presents an insightful graph of DOE interactions in a context devoid of migration, offering a clear view of how various factor combinations impact system availability in Edge Computing environments.

In Figure 9a, the graph underscores the system’s sensitivity to changes in the MTTF of the Edge component relative to the MTTF of the Network. A significant observation here is that an elevation in the Edge’s MTTF to 157.0 prompts a slight increase in the Network’s MTTF, from 95.0 to 95.3. This shift underscores the substantial influence that the Edge’s MTTF exerts on overall system availability.

Figure 9b examines the interplay between the MTTF of the physical RSU and that of its logical counterpart. It is observed that augmenting the reliability of the logical part of the RSU positively influences the MTTF of the physical RSU. This relationship highlights the criticality of integrating these factors to enhance the overall system performance.

Lastly, Figure 9c delves into the interaction between the MTTR and the MTTF of the physical RSU. An increase in the MTTR of the physical RSU is shown to improve the MTTF. However, an increase in MTTF does not significantly impact the MTTR. This finding accentuates the importance of a balanced approach to managing failure and repair times, as this balance is key to optimizing system availability.

Together, these insights from Figure 9 emphasize the complex nature of factor interdependencies in Edge Computing systems, particularly in scenarios where migration is not an option. Understanding these relationships is crucial for the strategic planning and implementation of measures aimed at enhancing the reliability and efficiency of such systems.

## 6. Case Study

In this segment, the paper delineates the findings from the analytical evaluation of the models introduced herein. This evaluation encompasses a comprehensive assessment of both the availability and reliability metrics for all the models under consideration. Table 6 provides a detailed account of the parameter values assigned to various system components, with these values meticulously sourced from extant scholarly publications [40,42,43,44]. The parameters detailed include those pertaining to the RSU group, the MTTF and MTTR for the Edge component, as well as the failure and repair durations associated with the network. Additionally, the table specifies the initial values for the tokens employed in the system, a critical element in the modeling process.

**Figure 10:**
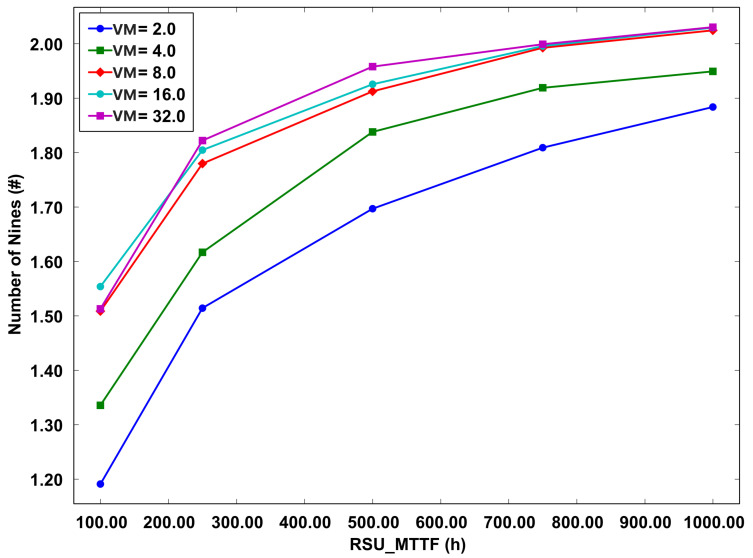
Availability model with migration.

The graph depicting system availability with migration, as shown in Figure 10, delineates a non-linear association between the quantity of VMs utilized and the consequent system availability. Notably, this graph exhibits a convergence of the availability metrics at specific VM counts, namely 8, 16, and 32. This overlapping of data lines suggests that the deployment of eight VMs is sufficient to assure the desired level of availability. Such an observation is pivotal in informing strategies for resource allocation and cost optimization. It implies that beyond a threshold of eight VMs, additional VMs do not significantly enhance system availability, thereby offering a pathway to maximize resource efficiency. This efficient allocation of VMs, without compromising the operational efficacy of the system, is essential in balancing cost effectiveness with system performance.

**Figure 11:**
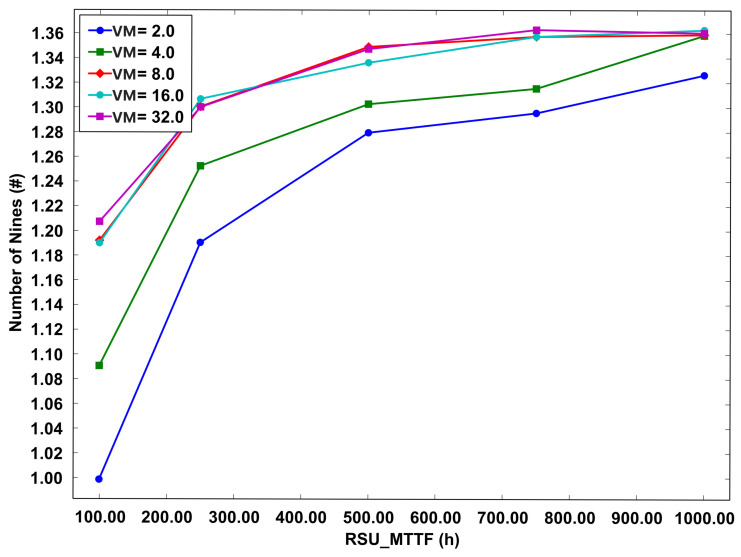
Availability chart—no migration model.

The research study includes Figure 11, which portrays the graph of system availability in scenarios where migration between RSUs is not implemented. This graph maintains consistency in the range of VMs as used in Figure 10, varying from 2 to 32 VMs in operation. The MTTF for these VMs is set between 100 and 1000 h. A critical observation from the results depicted in this graph is the comparative reduction in system availability when migration is not employed, as opposed to scenarios where migration is feasible.

Specifically, the graph shows that system availability commences at ‘1.00 nines’ with the operation of just 2 VMs, and it marginally escalates to ‘1.36 nines’ with the use of four VMs. This pattern of availability underscores the significance of migrating VMs between RSUs in enhancing system reliability. The ability to migrate VMs appears to be a crucial factor in achieving higher availability, as evidenced by the increase in the number of ‘nines’ in the availability metric. This insight highlights the importance of VM migration as a strategy to bolster the robustness and dependability of the system, especially in contexts where maintaining high availability is paramount. The study thus provides a compelling argument for incorporating VM migration between RSUs as a means to optimize system performance and reliability.

In the context of a system deploying four VMs with a MTTF set at 1000 h, it is feasible to achieve a level of system availability analogous to that obtained with a deployment of 32 VMs. This finding suggests that an augmentation in the number of VMs does not correspondingly result in a substantial increase in system availability. Particularly in the model excluding migration, the availability attributed to the logical component of the RSUs emerges as a predominant factor as discerned from the sensitivity analysis.

**Figure 12:**
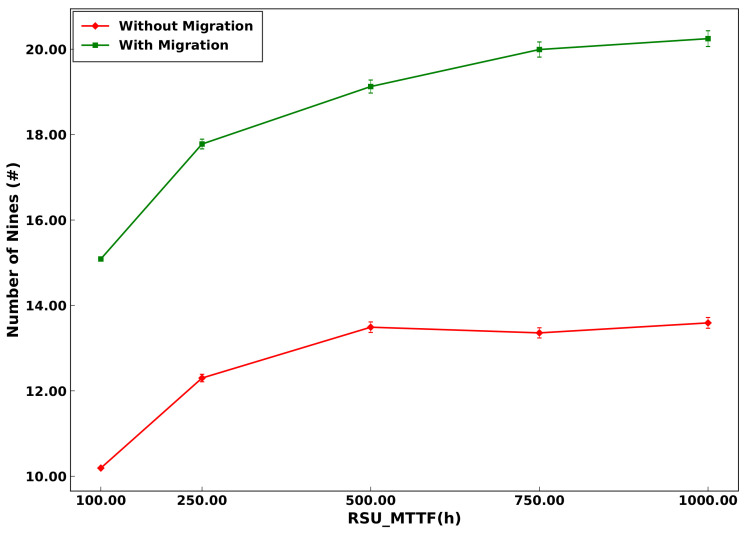
Comparison between the with-migration and without-migration models.

This distinction between the models with and without migration underscores the efficacy of the VM migration technique, especially in scenarios characterized by high demand. It accentuates the necessity of integrating VM migration into strategies for computing resource allocation. Figure 12 provides a comparative analysis, juxtaposing the average cases in scenarios of system operation with and without migration. This comparison elucidates the differential impacts of VM migration on system availability, thereby underscoring its critical role in the optimization of computing resources in demanding operational environments.

Figure 12 presents an analysis of system availability based on the failure time of RSUs to assess performance in configurations with and without VM migration. In scenarios without migration, the system demonstrates notable availability, achieving approximately 10.00 nines and increasing slightly to just over 14.00 nines when the MTTF is set at 100 h. Conversely, in configurations with migration, the availability shows a marked increase as the MTTF is extended, reaching an impressive 20.00 nines with an MTTF of 100 h. These results clearly indicate that migration exerts a positive influence on system availability, particularly in contexts characterized by extended MTTF periods.

**Figure 13:**
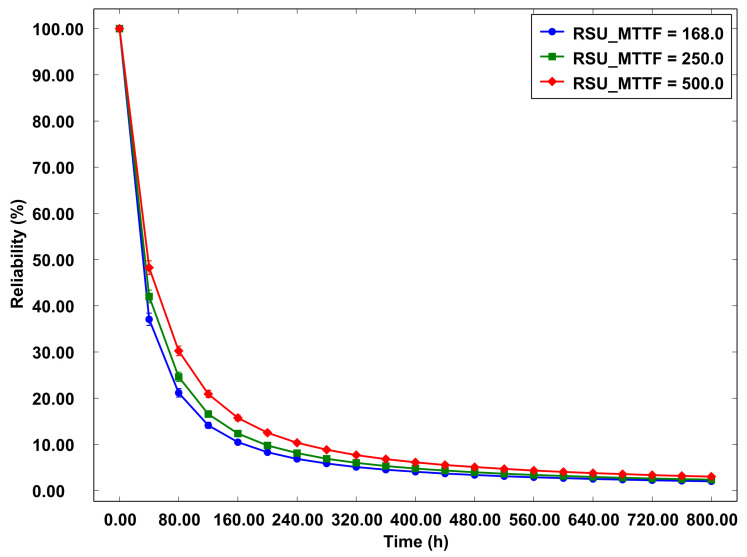
Reliability—physical part of the RSU.

Turning to Figure 13, the reliability graph for the physical component of the system illustrates the variance in the MTTF of the physical aspect of the RSU, with values spanning 168.0, 250.0, and 500.0 over a duration of 800 h. Reliability is a critical metric for evaluating the system’s capability to function consistently without failures and interruptions. The data portrayed in this graph establish a direct correlation between the MTTF of the physical component and the overall reliability of the system. This relationship indicates that an increase in the MTTF of the physical part is directly proportional to an enhancement in the system’s reliability. This insight is pivotal for understanding the impact of the physical component’s robustness on the overall operational stability of the system.

As the MTTF is extended, the system exhibits enhanced robustness and resilience, thereby diminishing the frequency of failures and bolstering reliable operations. Conversely, a system characterized by an MTTF of less than 168 h tends to be more vulnerable to failures and faces challenges in sustaining stable operations. This insight is invaluable for strategizing enhancements to the physical infrastructure of systems in Edge Computing environments. Effective planning in this context encompasses the implementation of both preventive and corrective strategies aimed at augmenting the reliability, quality, and availability of services delivered to end users.

**Figure 14:**
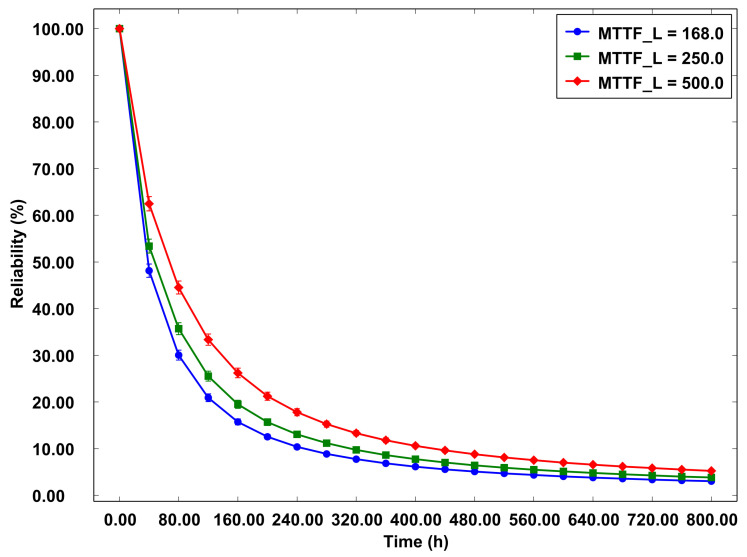
Reliability—logical part of the RSU.

Figure 14 displays the reliability graph for the logical component of the system, with the MTTF varying between 168.0, 250.0, and 500.0 over a span of 800 h. Assessing the MTTF of the logical part is vital to gauge its capacity to sustain adequate operational performance, particularly in Edge Computing contexts where uninterrupted availability is paramount. The data gleaned from this graph offer insights into the reliability trends of the system’s logical component relative to the MTTF of VMs.

It is observed that the system with the lowest MTTF of 168 h exhibits a decline in reliability before reaching 80 h of operation. This trend signifies a reduced capability of the system to maintain stability and remain free from failures over a shorter duration. In contrast, the system with the highest MTTF of 500 h demonstrates consistent reliability throughout the initial 80 h of operation, underscoring its superior ability to remain operational and reliable for an extended period before encountering declines in reliability. These findings are crucial in understanding the resilience of the logical component of the system and in guiding decisions related to the management and optimization of Edge Computing systems.

The analysis of the data underscores the heightened significance of the logical component in determining the system’s reliability, surpassing the influence of the physical part. Although both aspects are integral, the role of VMs, particularly their efficiency in terms of MTTF, emerges as a critical factor in ensuring uninterrupted system availability. The observation that a system with an elevated MTTF exhibits prolonged stable reliability prior to any decline in performance indicates that the efficacy and dependability of VMs are key determinants of system stability.

Consequently, it is imperative to place emphasis on strategies aimed at bolstering the reliability of the system’s logical aspect. This strategic focus encompasses several key measures:

- VM management policies: The development and implementation of comprehensive management policies for VMs are crucial. These policies should be designed to effectively handle resource allocation, scaling, and migration, thereby enhancing system performance.
- Performance monitoring: Rigorous and continuous monitoring of system performance is essential. This enables the early identification and resolution of potential issues, thereby maintaining the system’s operational integrity.
- Maintenance practices: The adoption of appropriate and systematic maintenance practices is vital in ensuring the optimal functioning of VMs. Regular maintenance activities, including updates and troubleshooting, are necessary for sustaining system health and efficiency.

Implementing these strategies will significantly enhance the availability and quality of the services rendered by the system. Such enhancements not only contribute to the system’s resilience and operational efficiency but also to the overall user experience in Edge Computing environments. Therefore, prioritizing improvements in the logical part of the system is not merely a technical imperative but a strategic approach to achieving superior service delivery.

## 7. Conclusions

This research meticulously analyzed system availability within networks of RSUs using SPN and the DOE methodology, focusing on environments with and without VM migration. The findings successfully met the study’s objectives, providing insightful revelations about the impact of critical factors on system performance in both migration scenarios. A significant aspect highlighted by the study is the critical role of VM migration in VANETs, which proves to be fundamental in optimizing system availability. This process not only ensures a balanced distribution of workload but also substantially minimizes system downtime. The study identifies the MTTF of physical RSUs and the MTTR of the logical components of RSUs as pivotal determinants of system availability. These findings emphasize the necessity of enhancing these components to secure reliable and efficient operation in VANET contexts.

For future research directions, a more granular investigation of the identified factor interactions is suggested. This should include considering a broader range of variables and conducting empirical experiments in real-world environments to further validate and refine the proposed model. Additionally, exploring alternative approaches to VM migration and incorporating advanced load-balancing strategies are proposed as avenues for further study. These investigations are expected to provide deeper insights and enable additional improvements in system availability and performance, especially in the complex and evolving landscape of Edge Computing. This comprehensive approach to future research will not only enhance the understanding of system dynamics in VANETs but also contribute to the development of more resilient and efficient vehicular network systems. 

## Figures and Tables

**Table 2:** Description of the main components of the model.

Type	Components	Description
Places	*MIGRATE_UP* *MIGRATE_DW* *EDGE_UP* *EDGE_DW* *NET_UP* *NET_DW* *RSU_UP1*, *RSU_UP2*, *RSU_UP3* *RSU_DW1*, *RSU_DW2*, *RSU_DW3* *RSU_LOG_UP*1, *RSU_LOG_UP*2, *RSU_LOG_UP*3 *RSU_LOG_DW*1, *RSU_LOG_DW*2, *RSU_LOG_DW*3	Migration of VMs between RSUs is available Migration of VMs between RSUs is unavailable Data processing at the Edge is available Data processing at the Edge is unavailable The network connecting the RSUs is available The network connecting the RSUs is unavailable The physical RSUs are available The physical RSUs are unavailable The logical RSUs are available The logical RSUs are unavailable
Transitions	*E_MTTR* *E_MTTF* *NET_MTTR* *NET_MTTF* *RSU_MTTR1*, *RSU_MTTR2*, *RSU_MTTR3* *RSU_MTTF1*, *RSU_MTTF2*, *RSU_MTTF3* *LOG_MTTR1*, *LOG_MTTR2*, *LOG_MTTR3* *LOG_MTTF1*, *LOG_MTTF2*, *LOG_MTTF3* T1, T2, T3, T4	Represents the MTTR of the system’s Edge computing Represents the MTTF of the system’s Edge computing Represents the MTTR of the system’s network Represents the MTTF of the system’s network Represents the MTTR of the system’s RSUs Represents the MTTF of the system’s RSUs Represents the MTTR of the logical part of the RSUs Represents the MTTF of the logical part of the RSUs Transitions between RSUs in the system

**Table 3:** Description of model storage conditions.

Places	Transition	Condition
*RSU_UP1*, *RSU_UP2*	T2	IF(#*RSU_LOG_DW*2 =0): (N -(#*RSU_UP1*+#*RSU_DW1*)) ELSE(#*RSU_UP1*+(#*RSU_UP1*+#*RSU_UP2*+#*RSU_UP3*))
*RSU_UP2*, *RSU_UP3*	T4	IF(#*RSU_LOG_DW*3=0): (N-(#*RSU_UP2*+#*RSU_DW2*)) ELSE(#*RSU_UP2*+(#*RSU_UP1*+#*RSU_UP2*+#*RSU_UP3*))

**Table 4:** Design table.

Factor Name	Factor Description	Low Setting	High Setting
*EDGE_F*	Edge MTTF	125.892	157.365
*NET_F*	Network MTTF	83,220.0	104,025.0
*RSU_F*	MTTF Physical Part of RSU	500.0	750.0
*RSU_R*	MTTR Physical Part of RSU	2.0	3.0
*LOG_F*	MTTF Logical Part of RSU	168.0	210.0

**Table 5:** Combination table.

*EDGE_F*	*NET_F*	*RSU_F*	*RSU_R*	*LOG_F*	Availability (%)
125.89	83,220.00	500.00	2.00	168.00	98.35
125.89	83,220.00	500.00	2.00	210.00	97.29
125.89	83,220.00	500.00	3.00	168.00	97.76
125.89	83,220.00	500.00	3.00	210.00	97.41
125.89	83,220.00	750.00	2.00	168.00	98.80
125.89	83,220.00	750.00	2.00	210.00	98.44
125.89	83,220.00	750.00	3.00	168.00	97.93
125.89	83,220.00	750.00	3.00	210.00	98.20
125.89	104,025.00	500.00	2.00	168.00	97.82
125.89	104,025.00	500.00	2.00	210.00	98.02
125.89	104,025.00	500.00	3.00	168.00	98.09
125.89	104,025.00	500.00	3.00	210.00	97.77
125.89	104,025.00	750.00	2.00	168.00	98.62
125.89	104,025.00	750.00	2.00	210.00	98.69
125.89	104,025.00	750.00	3.00	168.00	98.04
125.89	104,025.00	750.00	3.00	210.00	98.61
157.36	83,220.00	500.00	2.00	168.00	97.80
157.36	83,220.00	500.00	2.00	210.00	98.09
157.36	83,220.00	500.00	3.00	168.00	97.16
157.36	83,220.00	500.00	3.00	210.00	97.30
157.36	83,220.00	750.00	2.00	168.00	98.50
157.36	83,220.00	750.00	2.00	210.00	98.71
157.36	83,220.00	750.00	3.00	168.00	98.07
157.36	83,220.00	750.00	3.00	210.00	98.50
157.36	104,025.00	500.00	2.00	168.00	98.16
157.36	104,025.00	500.00	2.00	210.00	98.02
157.36	104,025.00	500.00	3.00	168.00	97.01
157.36	104,025.00	500.00	3.00	210.00	97.92
157.36	104,025.00	750.00	2.00	168.00	99.02
157.36	104,025.00	750.00	2.00	210.00	98.62
157.36	104,025.00	750.00	3.00	168.00	98.06
157.36	104,025.00	750.00	3.00	210.00	98.16

**Table 6:** Model parameters.

Type	Component	Definition	Value
MTTF	*EDGE_MTTF* *NET_MTTF* *RSU_MTTF* *RSU_LOG_MTTF*	EGDE component failure time Network component failure time RSU component failure time RSU Logical component failure time	125.89284 83,220.0 500.0 168.0
MTTR	*EDGE_MTTR* *NET_MTTR* *RSU_MTTR* *RSU_LOG_MTTR*	EGDE component recovery time Network component recovery time RSU component recovery time RSU Logical component recovery time	0.913794 12.0 2.0 2..0
Variável	TOKENS *T_M*	Entity representing a state or resource Migration Time	2.0 0.083333

## Data Availability

Data are contained within the article.
